# A Deep Clinical and Biochemical Characterization of a Patient With Combined Malonic and Methylmalonic Aciduria (CMAMMA)

**DOI:** 10.1002/jmd2.70045

**Published:** 2025-09-28

**Authors:** Vincenza Gragnaniello, Alfonso Galderisi, Sara Tucci, Mara Doimo, Marianna Caterino, Christian Loro, Chiara Cazzorla, Margherita Ruoppolo, Leonardo Salviati, Alberto B. Burlina

**Affiliations:** ^1^ Department of Women's and Children's Health University of Padua Padua Italy; ^2^ Department of Pediatrics Yale University New Haven Connecticut USA; ^3^ Pharmacy Medical Center—University of Freiburg Freiburg Germany; ^4^ Clinical Genetics Unit, Department of Women and Children's Health University of Padua Padua Italy; ^5^ Department of Molecular Medicine and Medical Biotechnology University of Naples “Federico II” Naples Italy

**Keywords:** *ACSF3* gene, CMAMMA, hyperinsulinism, hypoglycemia, newborn screening, urinary organic acids

## Abstract

Combined malonic and methylmalonic aciduria (CMAMMA) is an inborn error of metabolism caused by a deficiency in mitochondrial malonyl‐CoA synthetase, the enzyme responsible for activating malonic acid (MA) to malonyl‐CoA, a precursor of lipoic acid. The clinical phenotype is highly heterogeneous, ranging from asymptomatic cases to severe neurological impairment. We describe a patient affected by CMAMMA. The patient presented in the neonatal period with hyperinsulinemic hypoglycemia. Urinary organic acid analysis revealed elevated levels of both malonic and methylmalonic acids. The diagnosis of CMAMMA was confirmed through molecular testing of the *ACSF3* gene. Levels of both lipoylated pyruvate dehydrogenase (PDH) and α‐ketoglutarate dehydrogenase (αKGDH) were decreased. Given the role of lipoic acid in regulating insulin secretion, the involvement of impaired mitochondrial lipoic acid biosynthesis in the clinical presentation of hyperinsulinemic hypoglycemia cannot be excluded. We describe a case of CMAMMA associated with hyperinsulinemic hypoglycemia. While a definitive association between CMAMMA and hyperinsulinism cannot be established based on a single case, the observed reduction in lipoic acid levels may suggest a mechanistic connection between the two disorders. We suggest considering urinary organic acid testing in patients with hyperinsulinism, especially when the cause is unknown.

**Summary**

- We describe a case of CMAMMA associated with hyperinsulinemic hypoglycemia.
- The reduction in mitochondrial synthesis of lipoic acid could play a role in the dysregulated insulin secretion, although a definitive association cannot be established based on a single case, and further studies are needed.

## 1 Introduction

Combined malonic and methylmalonic aciduria (CMAMMA) is an inborn error of metabolism caused by biallelic variants of the *ACSF3* gene (OMIM #614265). *ACSF3* encodes a malonyl‐CoA synthetase, which belongs to the acyl‐CoA synthetase family 3 and converts malonic (MA) and methylmalonic (MMA) acid into their respective CoA forms. Its deficiency leads to the accumulation of MA and MMA in organs and tissues [1, 2]. Since CoA forms do not accumulate, propionylcarnitine levels do not increase and the disease cannot be identified by newborn screening programs based on acylcarnitine assay on dried blood spots [3, 4].

Patients with variants in the *ACSF3* gene exhibit a highly heterogeneous clinical phenotype [5, 6, 7]. During the first years of life, clinical manifestations are unspecific, ranging from symptoms of intermediary metabolic disorder to failure to thrive or developmental delay. During adulthood, psychiatric disorders and neurological signs may appear, including seizures, memory loss, cognitive decline, and psychiatric disorders [7, 8]. Asymptomatic individuals have also been reported, suggesting that CMAMMA may be present as a benign condition in some carriers of the homozygous variants [2, 5]. This variability may be influenced by a variable penetrance of mutations affecting ACSF3 enzyme activity and the ability of other enzymes to partially compensate for ACSF3 function [9].

Here, we describe a patient affected by CMAMMA who presented with severe hyperinsulinemic hypoglycemia since the first days of life. In light of the identification of a potential novel component of the CMAMMA phenotype, we conducted an in‐depth clinical, biochemical, and genetic characterization to explore possible pathogenic mechanisms linking the two conditions.

## 2 Patient and Methods

### 2.1 Case Report

The patient, a female from Northern Italy of non‐Hispanic white ethnicity, was born at term to non‐consanguineous parents. The pregnancy was uneventful, with a physiological postnatal transition (birthweight 3460 g (69th percentile), length 52 cm (72nd percentile), and head circumference 36.5 cm (94th percentile)). Expanded neonatal screening was negative [3].

On the second day of life, the patient experienced a symptomatic episode of hypoglycemia (14 mg/dL), characterized by sweating, hypotonia, and reduced responsiveness. The critical sample was suggestive of hyperinsulinemic hypoglycemia (as shown in Table 1). Treatment was started first with intravenous continuous infusion of glucagon at 1 mg/kg/day, followed by diazoxide up to 20 mg/kg.

**TABLE 1:** Results of critical samples during hypoglycemia.

	Neonatal period	5.5 years
Blood glycemia (mg/dL)	14	49
Blood gas analysis	No acidosis	No acidosis
Ammonia (μmol/L)	43	38
Lactate (mmol/L)	1.5	1.2
Insulin (mU/L, nv 3.2–16)	25.8	8
Beta‐hydroxybutyrate (mmol/L, nv < 0.6)	0.16	0.65
Non‐esterified fatty acids (mEq/L, nv 0.1–0.6)	0.94	0.336
Urinary organic acid	MMA 172 mmol/mol creatinine; MA: increased (qualitative assay)	MMA 120.7 μmol/mol creatinine; MA: increased (qualitative assay)
Dried blood spot acylcarnitine	Normal profile	Normal profile

### 2.2. Analysis of Urinary Organic Acids

Urinary sample was used for creatinine determination and organic acid liquid/liquid extraction before GC–MS injection, as described previously [10]. The dosage of the main organic acids, such as MMA, was quantitative, by comparison with an internal standard; the assessment of other organic acids, such as MA, was qualitative.

### 2.3. Genetic Testing

Genetic analysis was performed on genomic DNA extracted from peripheral blood mononuclear cells (PBMC) monocytes. The entire coding regions and intron‐exon boundaries of the genes were first analyzed using the clinical exome TruSight One kit (Illumina) according to the manufacturer's protocol and run on an Illumina MiSeq sequencer device. In a second evaluation, the sample was analyzed with the whole exome sequencing (WES) Exome 2.0.2 kit Plus Comprehensive Exome Spike‐in (Twist Bioscience). Libraries were prepared according to the manufacturer's protocol and run on an Illumina NextSeq 2000 sequencer. Secondary and tertiary analyses were performed with Dragen 4.2.4 and Emedgene 35.0.3 software. A virtual panel for hyperinsulinism was applied (Table S1).

### 2.4. Protein Modeling, Conservation Analysis, and Prediction

Images were generated with PyMOL software (v. 3.1) using as a template the malonyl‐CoA ligase bound to malonyl‐CoA and AMP from *Streptomyces coelicolor* crystal structure (Protein Data Bank code 3NYR). Human ACSF3 homolog sequences were obtained from the NCBI Protein Database. Multiple alignment was performed with Multialin (v.5.4.1).

### 2.5. Western Blot Analysis

PBMC extracts were resuspended in RIPA buffer and, after lysis and protein heat‐denaturation, subjected to sodium dodecyl sulfate polyacrylamide gel electrophoresis. Proteins were transferred to a nitrocellulose membrane that was incubated with the specific antibody (#ab58724, antilipoic acid polyclonal antibody, Abcam, Cambridge, UK) and detected by chemiluminescence. Quantitative analysis of band intensity was performed using the Image Lab Software of Bio‐Rad (Hercules, California) (for details see Supplementary Materials).

## 3. Results

### 3.1. Diagnostic Pathway

Metabolic investigation during the acute episode revealed elevated levels of urinary MA and MMA (as shown in Table 1). Acylcarnitine profile on dried blood spot was normal.

Based on these results, a suspicion of CMAMMA was raised and molecular diagnostics confirmed the diagnosis, revealing compound heterozygosity in the *ACSF3* gene: c.1061G>A/p.Arg354Gln + c.1725delC/p.Ser576Hisfs*96. No other variants were found in genes known to be associated with hyperinsulinism using a Next Generation Sequencing Panel. The panel comprised 55 genes (listed in Table S1) reported in the literature to be associated with isolated or syndromic hyperinsulinism, including some whose potential role was only inferred from murine studies. Based on current knowledge, no alternative genetic cause of congenital hyperinsulinism could be established.

### 3.2. Predicted Pathogenicity of the Variants

**FIGURE 1:**
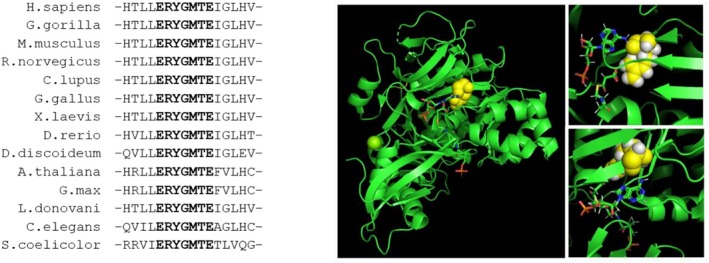
Effects of p.Arg354Gln variant. Multiple alignment of the region containing the active site of human ACSF3 protein and its homologs. The ERYGMTE motif is highlighted (A). Structure of the ACSF3 homolog from *Streptomyces coelicolor* showing the location of residue Arg354 (highlighted in yellow) in close proximity with the Malonyl‐CoA and AMP molecules (B). Images were generated with PyMOL software (v 3.1).

p.Arg354Gln variant was not previously reported. Arg354 is highly conserved throughout evolution (Figure 1, S1, and S2) and is located within the active site of the protein containing the ERYGMTE motif. Molecular modeling on the Malonyl‐CoA Ligase homolog from *S. coelicolor* reveals that Arg354 is located in a structured domain that accommodates the malonyl‐coenzyme A and the adenosine monophosphate (AMP) molecules. Variant c.1061G>A causes the substitution of the positively charged Arginine residue with the polar neutral amino acid Glutamine (p.Arg354Gln).

AlphaMissense predicts a likely pathogenic effect for this change (score 0.573—range of pathogenicity > 0.564). In vitro studies on the recombinant ACSF3 protein showed that substitution of arginine in position 354 with different amino acid residues disrupts the enzyme activity, supporting the hypothesis that Arg354 is involved in substrate binding.

Variation c.1725delC is a frameshift deletion that causes the formation of a premature stop codon in a gene where loss of function is a known mechanism of disease.

### 3.3. Effect of ACSF3 Mutations on Lipoilation Degree and Treatment With Medium Chain Triglycerides (MCT) and Lipoic Acid

**FIGURE 2:**
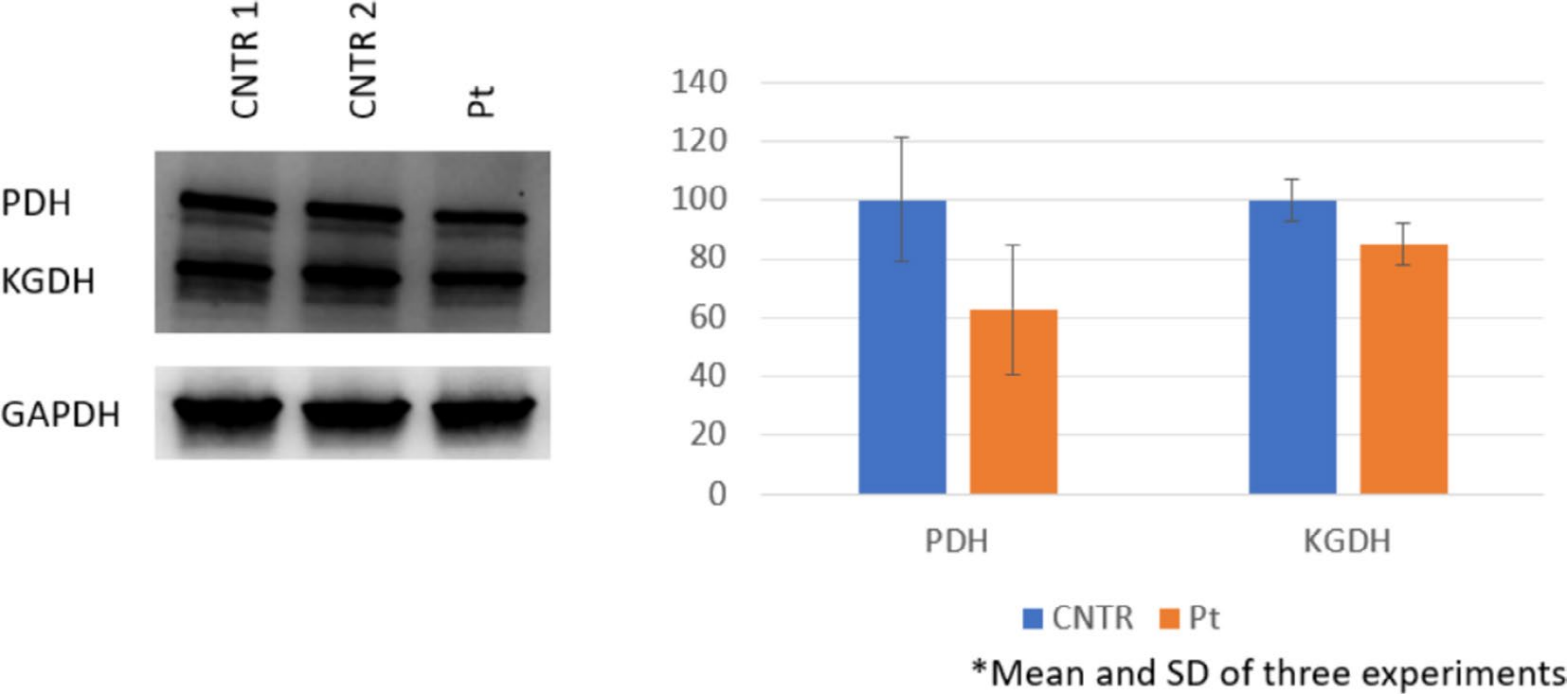
Western blot analysis and quantification of lipoic acid covalent bound to pyruvate dehydrogenase (PDH 70 kDa) and α‐Ketoglutarate dehydrogenase (α‐KGDH 55 kDa). Reduced lipoylation degree in PBMC of our patient compared to two controls matched for sex and age.

To evaluate the severity of the functional impairment of the ACSF3 enzyme, we investigated its lipoylation degree. Western blot analysis was performed on PBMCs using an antibody that specifically recognizes lipoic acid bound to proteins. As shown in Figure 2, we observed a tendency toward lower lipoylation degree in both pyruvate dehydrogenase (PDH) and alpha‐ketoglutarate dehydrogenase (αKGDH) (37% PDH—SEM 13%, 83% αKGDH, SEM 4%, mean of three experiments).

## 4. Follow‐Up

While on diazoxide treatment (20 mg/kg/day), the patient did not experience any hypoglycemic episodes. Two attempts to switch to octreotide (25 μg/kg/day) were unsuccessful due to the recurrence of episodes of hypoglycemia. An abdominal ultrasound performed at the age of 6 months revealed moderate hepatomegaly with fatty liver, which was confirmed during follow‐up at 2 and 5 years of age. Ophthalmologic and hearing screenings were normal, as were brain MRI and EEG. Psychomotor development was adequate for age (Bayley III at 1.5 years: cognitive 100, language 89, motor 82; WPPSI III at 2.5 years: IQ 114; at 5.5 years: IQ 103).

At 5.5 years of age, during a febrile intercurrent disease, the patient was admitted due to symptoms suggestive of hypoglycemia at home. During the admission, hypoglycemic hyperinsulinism was confirmed (49 mg/dL with non‐suppressed insulin concentrations). Therapy with diazoxide (15 mg/kg) allowed for good glycemic control outside of this infectious episode.

Due to impaired lipoylation of mitochondrial proteins, treatment with MCT (1 g/kg/day) was attempted, followed by lipoic acid at 200 mg twice daily (25 mg/kg/day). Unfortunately, these interventions did not improve the lipoylation degree (Figure S3).

## 5. Discussion

Neonatal hypoglycemia may result from a combination of maternal and neonatal factors [11, 12]. Based on current guidelines, it has been estimated that at least 25% of infants who are currently considered not at risk develop transient or persistent neonatal hypoglycemia during the first week of life [13]. The diagnostic workup is based on findings from critical samples collected during the episode, and the contemporary presence or absence of hyperinsulinemia drives the diagnostic pathway [11]. Herein we identify, for the first time, CMAMMA in a patient with neonatal hypoglycemia. The absence of acidosis, reduced levels of beta‐hydroxybutyrate and non‐esterified fatty acids, and the response to glucagon infusion led to the diagnosis of hyperinsulinism.

The detection of increased urinary MA and MMA, despite a normal acylcarnitine profile, raised suspicion of CMAMMA, which is consistent with the negative newborn screening. Genetic testing confirmed the diagnosis, revealing previously undescribed variants. Prediction and biochemical studies confirmed their pathogenicity in our patient.

Based on biochemical evidence and current knowledge about the regulation of insulin secretion [14, 15], a potential disease‐causing mechanism for underlying hyperinsulinism may involve a defect in intracellular lipoic acid availability that we demonstrated in our patient.

Indeed, the disease is characterized by impaired mitochondrial fatty acid synthesis due to a deficiency of intramitochondrial malonyl‐CoA [16]. Malonyl‐CoA is the essential precursor for mitochondrial de novo fatty acid biosynthesis (mtFAS), forming octanoyl‐ACP (an octanoyl moiety bound to acyl‐carrier protein), which is necessary for mitochondrial lipoic acid synthesis [1, 2]. There is no agreement on the reduction in lipoic acid synthesis in CMAMMA in vitro studies [16, 17]. The variability in lipoylation may be due to different residual enzyme activities [9], as demonstrated by studies on RNAi‐mediated knockdown of ACSF3 in HEK293 cell lines showing a protein lipoylation defect only when ACSF3 transcript levels are reduced to less than 40% of WT [18, 19]. These factors may also explain the wide clinical heterogeneity observed in CMAMMA. The reduced lipoylation levels observed in our patient may be due to particularly impaired enzyme activity, given the compound heterozygosity of a null variant with a missense variant involving the active site.

This finding is relevant because the lipoic acid, reduced in our patient, is also an inhibitor of both acute and chronic insulin secretion [14, 15]. Therefore, a lipoic acid defect could contribute to uncontrolled insulin secretion in our CMAMMA patient.

We attempted lipoic acid supplementation in our patient but observed no improvements in mitochondrial protein lipoylation. This finding supports previous in vitro evidence showing that exogenous lipoic acid supplementation fails to restore mitochondrial function, highlighting the essential role of endogenous biosynthesis of C8 via the mtFAS pathway [20, 21]. We also tried supplementation with medium‐chain fatty acids, as a C8 source, but it did not improve the lipoylation degree of the analyzed PBMCs of the patient.

In conclusion, we describe a case of CMAMMA associated with hyperinsulinemic hypoglycemia.

Although the observed reduction in lipoic acid levels may suggest a possible mechanistic connection between the two disorders, a clear association between CMAMMA and hyperinsulinism cannot be established based on a single case, and the two clinical conditions could be independent.

As the disease cannot be identified through neonatal screening on dried blood spots, we suggest considering urinary organic acid testing in patients with hyperinsulinism, especially when the cause is unknown. Further studies in murine models are needed to confirm the exact pathogenesis of this possible new manifestation and to develop targeted therapies.

## Author Contributions

**Vincenza Gragnaniello:** conceptualization, data curation, formal analysis, investigation, project administration, visualization, roles/writing – original draft. **Alfonso Galderisi:** conceptualization, writing – review and editing. **Sara Tucci:** conceptualization, formal analysis, visualization, writing – review and editing. **Mara Doimo:** data curation, formal analysis, investigation, visualization. **Marianna Caterino:** formal analysis, investigation. **Christian Loro:** investigation. **Chiara Cazzorla:** investigation. **Margherita Ruoppolo:** validation. **Leonardo Salviati:** validation. **Alberto B. Burlina:** conceptualization, supervision, validation, writing – review and editing.

## Consent

Written informed consent was obtained from the patient's parents for publication of this case report.

## Conflicts of Interest

The authors declare no conflicts of interest.

## Supporting information


**Data S1:** jmd270045‐sup‐0001‐Supinfo.docx.

## Data Availability

The data that support the findings of this study are available on request from the corresponding author. The data are not publicly available due to privacy or ethical restrictions.
