# Supplementary material for: A Deep Clinical and Biochemical Characterization of a Patient With Combined Malonic and Methylmalonic Aciduria (CMAMMA)
Source: JIMD Rep. 2025 Sep 28;66(6):e70045. doi: 10.1002/jmd2.70045 (PMC12476969; doi:10.1002/jmd2.70045)
Supplement: Supplementary file 1 — Data S1: jmd270045‐sup‐0001‐Supinfo.docx. [file JMD2-66-e70045-s001.docx]

**Supplementary materials**

**Western blot analysis**

Blood samples were centrifuged at 3000 rpm, and the cell pellet was processed to isolate peripheral blood mononuclear cells (PBMCs) using density gradient centrifugation on Ficoll (Cytiva™, Uppsala, Sweden).

PBMC extracts were resuspended in RIPA buffer, supplemented with 1% protease and phosphatase inhibitors (Thermo Fisher Scientific, Waltham, Massachusetts). The extracts were disrupted by vortexing and centrifuged at 1300 rpm for 30 min at 4 °C. Protein concentration was measured using the BCA (bicinchoninic acid) assay according to the manufacturer’s instructions (Pierce™ BCA Protein Assay Kit; Thermo Fisher, Waltham, Massachusetts). Equal amounts (20 µg of protein) of extracts were heat-denatured for 5 min at 95 °C and subjected to sodium dodecyl sulfate polyacrylamide gel electrophoresis (4–15% polyacrylamide). Proteins were transferred to a nitrocellulose membrane, which was incubated with blocking solution (Everyblot, Bio-Rad) for 5 min. Then, it was incubated with the primary antibody (anti-lipoic acid polyclonal antibody; #ab58724, Abcam, Cambridge, UK) overnight at 4°C and subsequently with secondary antibodies (horseradish peroxidase HRP conjugated anti-rabbit IgG) for 1 h. Immunoreactive proteins were detected by chemiluminescence (ECL, Bio-Rad). The GAPDH peptide was used to allow comparison of different samples. All reagents (blocking solution, precast gels, nitrocellulose membranes, TBS Tween 20, ECL) and instruments (Transblot, Chemidoc) were from Bio-Rad (Hercules, California). Primary antibodies were from Abcam and used at a dilution of 1:1000. Secondary antibodies were from Bio-Rad and used at a dilution of 1:2000. Quantitative analysis of band intensity was performed using the Image Lab Software of Bio-Rad (Hercules, California).

**Sup. Figure 1:** Alignment of human ACSF3 and homologs from different species. The ERYGMTE motif containing the Arg354 residue is indicated with a black square. The sequences of human ACSF3’s homologs were obtained by the NCBI proteins database. Alignment was performed with Multialin software (v.5.4.1).


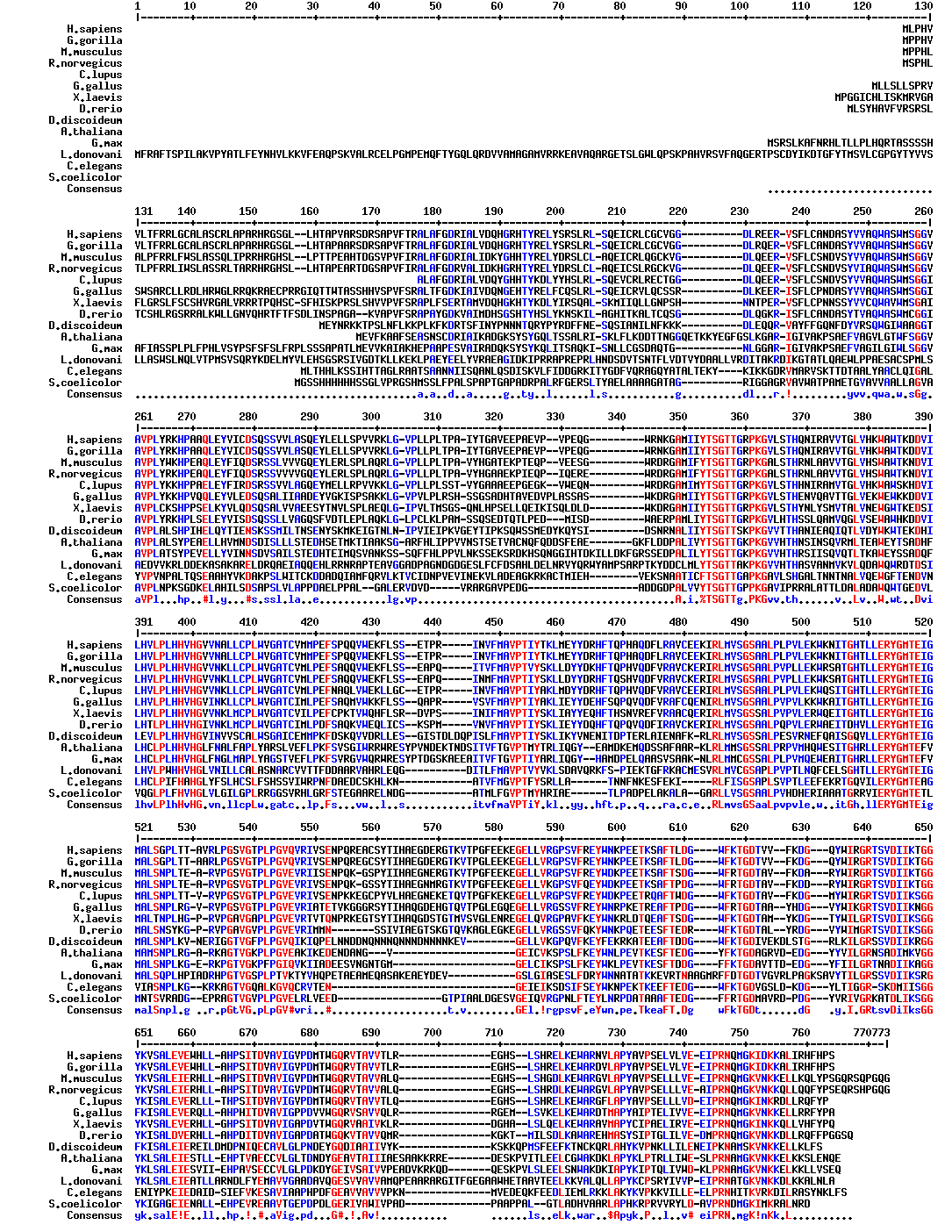


**Sup. figure 2:** BLAST alignment of human ACSF3 (up) and the Malonyl-CoA Ligase from *Streptomyces coelicor* (bottom) used for the molecular modelling.


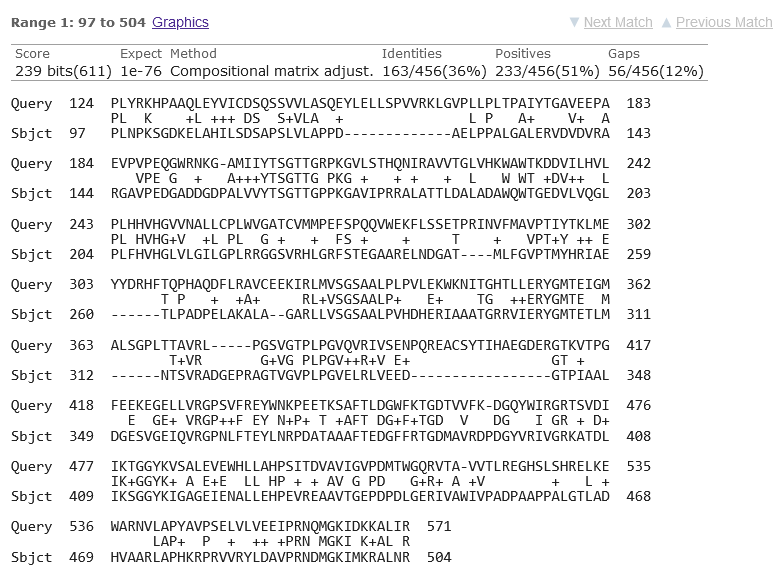


**Sup. Figure 3: Western blot analysis and quantification of lipoic acid covalent bound to pyruvate dehydrogenase (PDH 70 kDa) and α-Ketoglutarate dehydrogenase (α-KGDH 55 kDa).** Lipoylation degree did not improve after treatment with MCT (A) nor lipoic acid (B).


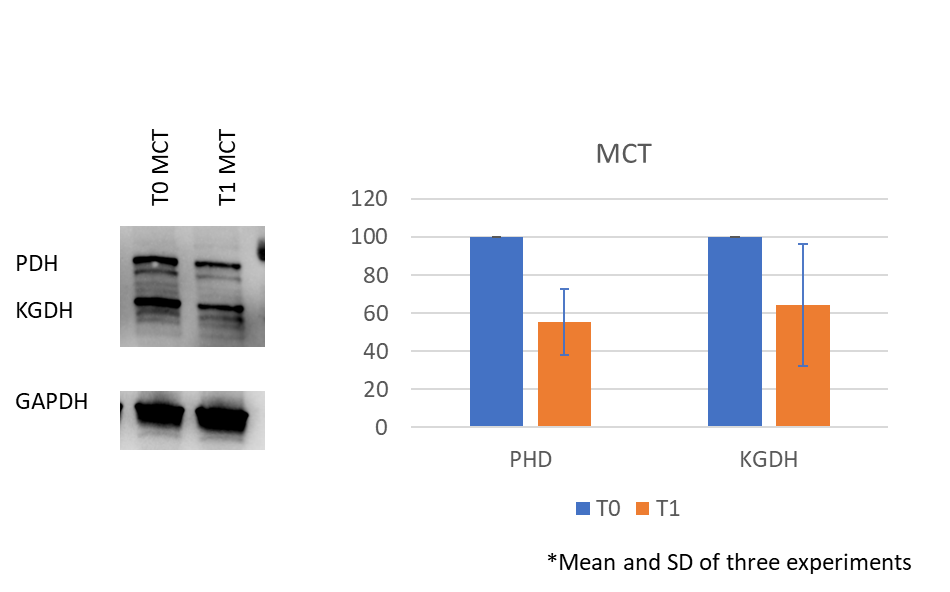


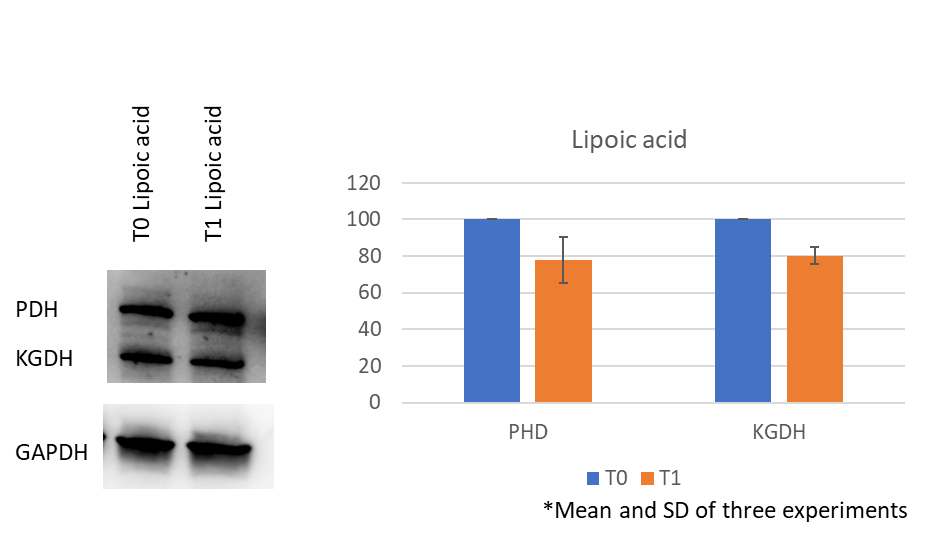


**Sup. Table 1:** List of genes included in the NGS panel for Hyperinsulinism.

| GENE | Inheritance | Phenotype | OMIM (#) |
| --- | --- | --- | --- |
| ABCC8 | AD/AR | Hyperinsulinemic hypoglycemia, familial, 1 | #256450 |
| GCK | AD | Hyperinsulinemic hypoglycemia, familial, 3 | #602485 |
| KCNJ11 | AD/AR | Hyperinsulinemic hypoglycemia, familial, 2 | #601820 |
| HADH | AR | Hyperinsulinemic hypoglycemia, familial, 4 | #609975 |
| INSR | AD | Hyperinsulinemic hypoglycemia, familial, 5 | #609968 |
| SLC16A1 | AD | Hyperinsulinemic hypoglycemia, familial, 7 | #610021 |
| SLC25A36 | AR | Hyperinsulinemic hypoglycemia, familial, 8 | #620211 |
| GLUD1 | AD | Hyperinsulinism-hyperammonemia syndrome | #606762 |
| KMT2D | AD | Kabuki syndrome | #147920 |
| KDM6A | XLD | Kabuki syndrome | #300867 |
| NSD1 | AD | SOTOS syndrome with hyperinsulinemic hypoglycemia | #117550 |
| MPI | AR | Congenital disorder of glycosylation, type Ib | #602579 |
| ALG3 | AR | Congenital disorder of glycosylation, type Id | #601110 |
| STX5 | AR | Congenital disorder of glycosylation, type IIaa | #620454 |
| PGM1 | AR | Congenital disorder of glycosylation, type It | #614921 |
| ALG6 | AR | Congenital disorder of glycosylation, type Ic | #603147 |
| PMM2 | AR | Congenital disorder of glycosylation, type Ia | #212065 |
| TRMT10A | AR | Microcephaly, short stature, and impaired glucose metabolism 1 | #616033 |
| YARS1 | AR | Infantile-onset multisystem neurologic, endocrine, and pancreatic disease 2 | #619418 |
| EIF2S3 | XLR | MEHMO syndrome (hyperinsulinemic hypoglycemia and postprandial hyperglycemia) | #300148 |
| KCNQ1OT1 | AD | Beckwith-Wiedemann syndrome | #130650 |
| CDKN1C | AD | Beckwith-Wiedemann syndrome | *600856 |
| PTEN | AD | Cowden syndrome | #158350 |
| MAGEL2 | AD | Schaaf-Yang syndrome 1 | #615547 |
| DNAJC3 | AR | Ataxia, combined cerebellar and peripheral, with hearing loss and diabetes mellitus | #616192 |
| CREBBP | AD | Rubinstein-Taybi syndrome 1 | #180849 |
| EP300 | AD | Rubinstein-Taybi syndrome 2 | #613684 |
| GPC3 | XLR | Simpson-Golabi-Behmel syndrome, type 1 | #312870 |
| USH1C | AR | Usher syndrome, type 1C | #276904 |
| CACNA1D | AD | Primary aldosteronism, seizures, and neurologic abnormalities | #615474 |
| HRAS | AD | Costello Syndrome | #218040 |
| KCNQ1 | AD | Long QT Syndrome | #192500 |
| HNF4A | AD | MODY, type I-hyperinsulinemia at birth | #125850 |
| HNF1A | AD/AR | MODY, type III | #600496 |
| AKT2 | AD | Diabetes mellitus type II | *164731 |
| MAFA | AD | Insulinomatosis and diabetes mellitus | #610303 |
| PPARG | AD | Insulin resistance, severe, digenic | #604367 |
| IRS2 | AD | {Diabetes mellitus, noninsulin-dependent} | *600797 |
| IRS1 | AD | {Type 2 diabetes mellitus, susceptibility to} | *147545 |
| ADRB3 | AR/AD | {Obesity, susceptibility to} | *109691 |
| UCP2 |  | {Obesity, susceptibility to, BMIQ4} | #607447 |
| ENPP1 | AD | {Obesity, susceptibility to} | *173335 |
| INS | AD | Association with hyperinsulinemia | *604115 |
| HK1 | AD | Possible association to Hyperinsulinemic hypoglycemia (PMID: 23859901) | *142600 |
| CACNA1C | AD | Possible association to Hyperinsulinemic hypoglycemia (PMID: 35897673) | *114205 |
| GCG |  | Possible gene association due to mouse study, not disease related | *138030 |
| SIRT1 |  | Possible gene association due to mouse study, not disease related | *604479 |
| PPARGC1A |  | Possible gene association due to mouse study, not disease related | *604517 |
| PDPK1 |  | Possible gene association due to mouse study, not disease related | *605213 |
| SOX6 |  | Possible gene association due to mouse study | *607257 |
| UCN2 |  | Possible gene association due to mouse study, not disease related | *605902 |
| APOM |  | Possible gene association due to mouse study, not disease related | *606907 |
| PPARGC1B |  | Possible gene association due to mouse study, not disease related | *608886 |
| FOXA2 |  | Possible gene association due to mouse study, not disease related | *600288 |
| VEGFA |  | Possible gene association due to mouse study, not disease related | *192240 |
